# Myxozoan infection in thinlip mullet *Chelon ramada* (Mugiliformes: Mugilidae) in the Sea of Galilee

**DOI:** 10.1038/s41598-022-13215-z

**Published:** 2022-06-16

**Authors:** Aditya Gupta, Michal Haddas-Sasson, Kfir Gayer, Dorothée Huchon

**Affiliations:** 1 School of Zoology, George S. Wise Faculty of Life Sciences, Tel Aviv University, 6997801 Tel-Aviv, Israel; 2 Steinhardt Natural History Museum, Tel Aviv University, 6997801 Tel-Aviv, Israel

**Keywords:** Parasite genetics, Pathogens, Molecular biology, Ichthyology

## Abstract

Mullets (Mugilidae) are economically important fish in Israel. Two species of mugilids (i.e., the thinlip mullet *Chelon ramada* and the flathead grey mullet *Mugil cephalus*) have been stocked in the Sea of Galilee (Lake Kinneret) in order to increase fishermen’s income and lake water quality. These catadromous species do not reproduce in the lake, consequently, fingerlings have been introduced every year since 1958. Few additional mugilid species have been introduced unintentionally together with these two species, including *C. labrosus*. Following a survey of myxozoan infections in the Sea of Galilee, we described *Myxobolus pupkoi* n. sp. infecting the gill arches of *C. labrosus*, and reported *Myxobolus exiguus* from visceral peritoneum and gall bladder of *C. ramada*. Our study indicates that the parasites infecting *C. ramada* and *C. labrosus* belong to a lineage of myxozoans infecting mugilids. This result suggests that the infection took place in the Mediterranean Sea, where the fingerlings were caught, before their introduction into the Sea of Galilee. Since 2018 only farm-raised fingerlings have been introduced. We thus recommend to closely monitor the presence of these parasites in the future to determine if the presence of parasites disappear with the introduction of farm-raised fingerlings.

## Introduction

Mugilidae is a large fish family that includes 17 genera and 80 species. Mullets are present worldwide, mainly in coastal shallow marine and brackish waters of the tropical and temperate regions. They are an appreciated food resource with high commercial value^1^. In Israel, two mullet species (*Mugil cephalus* Linnaeus, 1758 and *Chelon ramada* Risso, 1827) have been artificially introduced in large numbers since 1958 in the Sea of Galilee (Lake Kinneret), the largest freshwater body in the Levant. The fish were introduced in order to lower the algal population and increase the annual fish yield^2,3^. Specifically, until 2018, mugilid fingerlings collected along the Mediterranean coast and in coastal streams were introduced into the Sea of Galilee after a 1–2 days stay in freshwater ponds for adaptation^2,4^. From 1960 to 2018, about one million fingerlings of *M. cephalus* and *C. ramada* were introduced every year into the Sea of Galilee^5^. Since 2018, the number of fish introduced annually has been reduced to only a few tens of thousands of *M. cephalus* fingerlings, originating from breeding farms (Dr. Menachem Goren, Tel-Aviv University, personal communication).

Myxozoans are one of the main parasitic lineage infecting mullets^6–8^. Myxozoans form a clade of highly-reduced, microscopic, endoparasites Cnidaria. They have complex life-cycles involving two hosts, usually a fish (the intermediate host) and an annelid (the definite host)^9^. Over 90 myxozoan species have been reported to infect mullets^10,11^. The majority of myxozoans that parasitize mullets belong to the family Myxobolidae, including 50 species of the genus *Myxobolus* Butschli, 1882^12,13^. Interestingly, while myxobolids are mainly freshwater species, both marine and freshwater myxobolids are known to infect mugilids^10^.

During a survey of myxozoans infecting fish from the Sea of Galilee, we found myxozoan infections in the gall bladder, visceral peritoneum and gill arches of a few *C. ramada* specimen. A taxonomic and molecular study was performed to identify the parasites and determine whether they had a marine or freshwater origin. While Myxozoa are often host specific, a freshwater origin of the parasites could indicate a host switch. In contrast, a marine origin, would indicate that the fish and their parasites were co-introduced to the Sea of Galilee.

## Results

Myxozoan parasites were searched for in ten *M. cephalus* and twenty-three *C. ramada* specimens sampled in the Sea of Galilee on November 2020. While no myxozoan parasite was observed in *M. cephalus*, plasmodia were found in the gall bladder, in the visceral peritoneum lining the intestine and the gill arches of four *C. ramada* specimen. Each of the four fish was infected by a single parasite. No infection was found in other organs such as kidney, liver, eyes, brain, fins, muscles, and scales. The 18S rRNA sequences from the plasmodia from gall bladder and visceral peritoneum were identical to *M. exiguus* reported from Portugal (sequence accession number: MH236070)^14^, except for three insertions in the last 7 bp of the sequence, which most probably correspond to sequencing errors since most are located in the primer region. The parasite obtained from gill arches did not match any other myxosporean sequence available in the NCBI database.

### *Myxobolus exiguus* Thélohan, 1895 from the Sea of Galilee

**Plasmodia**: Small plasmodia (about 0.5 mm), visible with naked eye, rounded, creamish white, freely floating in the bile or present in the visceral peritoneum lining the intestine, containing 300–500 myxospores per plasmodium.

**Myxospore**: The morphological data are based on spores isolated from two plasmodia, one originating from the gall bladder and another from the visceral peritoneum. Few morphological differences were found between the spores of *M. exiguus* infecting the visceral peritoneum and the gall bladder of *C. ramada*.

**Figure 1:**
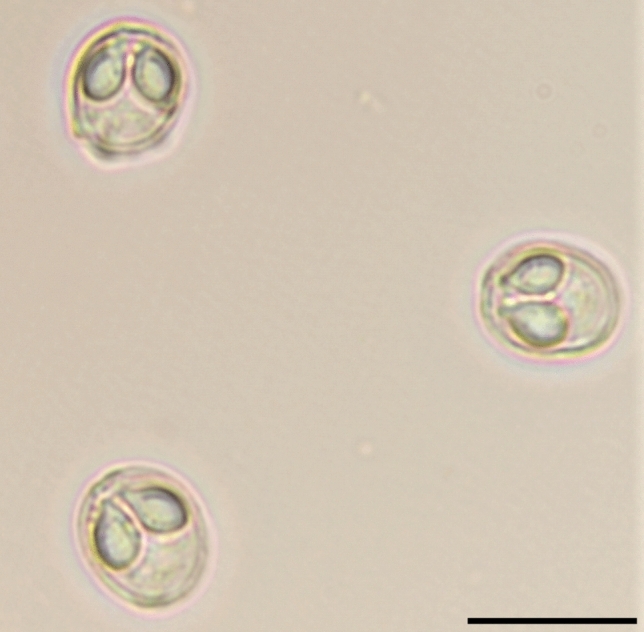
*Myxobolus exiguus* Thélohan, 1895 parasite of the visceral peritoneum and gall bladder of *Chelon ramada* (frontal view). Scale bar: 10 μm.

Mature gall bladder myxospores broadly oval to sub-spherical in valvular view and ellipsoidal in sutural view (Fig. 1). They measured 7.80 ± 0.34 (7.15–8.64) µm in length and 6.60 ± 0.22 (6.30–6.95) µm in width (n = 12). Those from the visceral peritoneum were smaller, measured 6.63 ± 0.22 (6.15–7.04) µm in length and 6.01 ± 0.19 (5.50–6.40) µm in width (n = 12). Inter-capsular process absent; shell valve thick, measuring 0.57 ± 0.04 µm; sporoplasm finely granular filling the inter-capsular space presenting two nuclei of slightly different size measuring 1.25 ± 0.03 and 1.10 ± 0.02 µm for the larger one and the smaller one, respectively (Fig. 1; Table 1).

**Table 1:** Measurements (µm) and ratio of *Myxobolus exiguus* Thélohan, 1895 and *Myxobolus pupkoi* n. sp. (n = 12).

Characters	Mean values		Range		SD	
	*Myxobolus exiguus* Thélohan, 1895	*Myxobolus pupkoi* n. sp.	*Myxobolus exiguus* Thélohan, 1895	*Myxobolus pupkoi* n. sp.	*Myxobolus exiguus* Thélohan, 1895	*Myxobolus pupkoi* n. sp.
LS	7.80	5.84	7.15–8.64	5.67–6.14	0.35	0.13
WS	6.60	5.41	6.30–6.95	5.30–5.53	0.24	0.09
LPC	3.79	2.44	3.54–3.97	2.25–2.73	0.13	0.20
WPC	2.54	1.34	2.41–2.67	1.14–1.80	0.07	0.19
NC	–	–	3–4	4–5	–	–
LS/WS	1.18	1.079	1.12–1.28	1.02–1.11	0.046	0.025
Sutural fold	–	–	Absent	8–9	–	–

*LS* length of spore, *WS* width of spore, *LPC* length of polar capsule, *WPC* width of polar capsule, *NC* number of coils, *SD* standard deviation.

Polar capsules pyriform and bottle-necked, equal in size and arranged side by side with two prominent pores at the anterior side of the myxospore measuring 3.79 ± 0.12 (3.54–3.97) µm in length and 2.54 ± 0.07 (2.41–2.67) µm in width (from gall bladder); 3.41 ± 0.11 (3.04–3.85) µm in length and 2.16 ± 0.05 (1.95–2.30) µm in width (from the visceral peritoneum). Polar tubules form 3–4 coils while inside the capsule (Fig. 1; Table 1).

**Host:** *Chelon ramada* (Risso, 1827), vern. thinlip mullet, Family: Mugilidae.

**Locality:** Sea of Galilee (31° 49′ N, 35° 38′ E), Israel.

**Material deposited:** Four slides with stained myxospores (SMNHTAU-AP-50-53) have been deposited in the parasite collection of the Steinhardt Museum of Natural History, Tel Aviv University, Israel.

**Infection site:** Gall bladder bile and visceral peritoneum.

Because of fish misidentification the prevalence of infection cannot be computed.

**Clinical signals:** Whitish patches floating inside the gall bladder and in the intestine visceral peritoneum.

**Sequences deposited:** Two identical sequences were deposited. GenBank accession numbers OM065835 (2017 bp—gall bladder) and OL604467 (2017 bp—visceral peritoneum).

**Remarks**: The present observations on *M. exiguus*^15^ show that the spore size is smaller in the Israeli specimen than previously described^14^. Previous work reported *M. exiguus*^15^ from the visceral peritoneum of *C. ramada, C. auratus, C. saliens, C. labrosus* and *M. cephalus* from France, Tunisia and Portugal. A new locality—the Sea of Galilee, Israel—and a new tissue—the gall bladder—are here recorded for this parasite.

### *Myxobolus pupkoi* n. sp

**Plasmodia:** Small histozoic plasmodia, visible with naked eye, rounded, creamish white (about 1.0 mm in diameter), a single infected gill arch per fish with 2–4 plasmodia, 300–500 myxospores per plasmodium. Gills pale and mucous laden.

**Figure 2:**
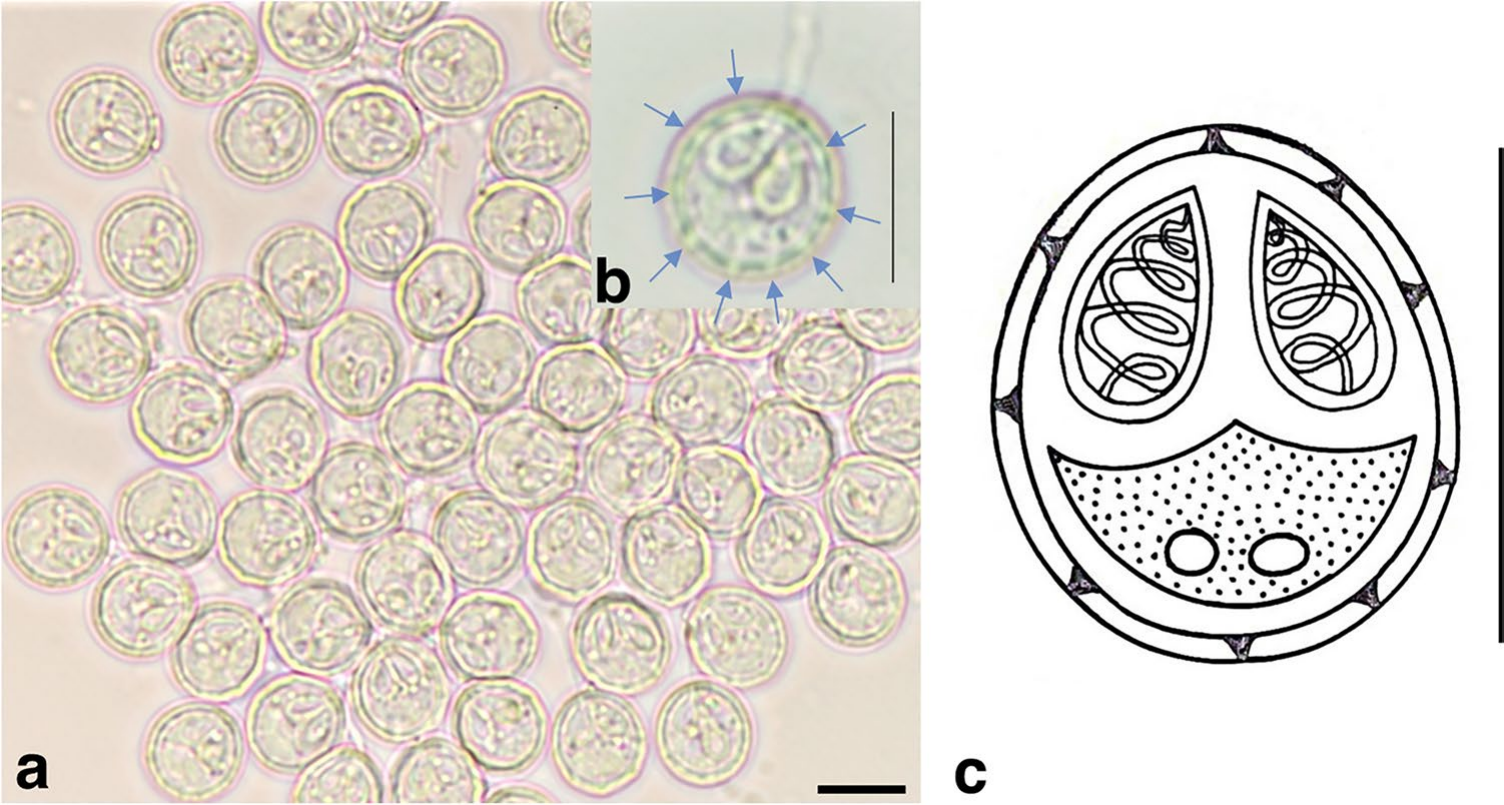
*Myxobolus pupkoi* n. sp. parasite of the gill arch of *Chelon labrosus*. (**a**, **b**) Photomicrographs of mature myxospores (frontal view). Note: arrows indicating sutural folds over the myxospore body. (**c**) Schematic drawing of a myxospore. Scale bars: 5 μm.

**Myxospore:** The morphological data are based on the observation of myxospores isolated from a single plasmodium. Mature myxospores sub-spherical in valvular view and ellipsoidal in sutural view, measuring 5.84 ± 0.13 (5.67–6.14) µm in length and 5.41 ± 0.09 (5.30–5.53) µm in width (n = 12). Polar capsules pyriform, equal in size, and located at the anterior half of the myxospore measuring 2.44 ± 0.19 (2.25–2.73) µm in length and 1.34 ± 0.18 (1.14–1.80) µm wide (n = 12). Polar tubules making 4–5 coils while inside the capsule and measuring 7–10 µm when extruded; inter-capsular process absent; 8–9 sutural folds on the ring of the myxospore; sutural line thick, measuring 0.65 ± 0.03 µm and slightly curved; sporoplasm finely granular with two nuclei of similar size measuring 0.95 ± 0.04 µm, filling most of the extracapsular cavity (Fig. 2; Table 1).

**Type host:** *Chelon labrosus* (Risso, 1827), vern. thicklip grey mullet, Family: Mugilidae.

**Type locality:** Sea of Galilee (31° 49′ N, 35° 38′ E), Israel.

**Material:** Two slides with stained myxospores (SMNHTAU-AP-48-49) have been deposited in the parasite collection of the Steinhardt Museum of Natural History, Tel Aviv University, Israel.

**Infection site:** Gill arch.

Because of fish misidentification the prevalence of infection cannot be computed.

**Clinical signals:** Whitish plasmodia on the gill arch.

**Sequence:** GenBank accession number OL605966 (2013 bp).

**Etymology:** The specific epithet “*pupkoi*” has been given in honor of Prof. Tal Pupko, an expert in molecular evolution and bioinformatics, for his endless support, and encouragements.

**Remarks**: For differential diagnosis, *M. pupkoi* n. sp. morphology, infection site and host have been compared with other *Myxobolus* species infecting members of the genus *Chelon*^10,15–23^ (Table 2).

**Table 2:** Comparative description of *Myxobolus pupkoi* n. sp. with myxobolid species infecting the genus *Chelon* (measurements in micrometer).

Species	Host	Site of infection	Locality	Myxospores	Polar capsule	No. of coils	Parietal folds	LS/WS ratio
*Myxobolus pupkoi* n. sp. (present study)	*Chelon labrosus*	Gill arch	Sea of Galilee, Israel	5.84 ± 0.13 × 5.41 ± 0.09	2.44 ± 0.20 × 1.34 ± 0.19	4–5	Present	1.07
*M. exiguus* (present study)	*C. ramada*	Gall bladder	Sea of Galilee, Israel	7.80 ± 0.35 × 6.60 ± 0.24	3.79 ± 0.13 × 2.54 ± 0.07	3–4	Absent	1.18
*M. exiguus* (present study)	*C. ramada*	Visceral peritoneum	Sea of Galilee, Israel	6.63 ± 0.25 × 6.01 ± 0.30	3.41 ± 0.19 × 2.16 ± 0.10	3–4	Absent	1.10
*M. adeli*^8^	*C. auratus*	Digestive tract, swim bladder, gills, muscle	Mediterranean Sea off Spain, Azov and Black Sea	6.2 ± 0.3 × 7.2 ± 0.3	3.1 ± 0.3 × 1.8 ± 0.2	4	Absent	0.86
*M. adiposus*^10^	*C. ramada*	Adipose tissue	River Minho, Portugal	9.1 ± 0.3 × 9.0 ± 0.3	4.6 ± 0.3 × 3.0 ± 0.3	6–7	Present	1.01
*M. cerveirensis*^10^	*C. ramada*	Intestine	River Minho, Portugal	8.1 ± 0.2 × 6.8 ± 0.2	4.2 ± 0.2 × 2.8 ± 0.2	4–5	Present	1.19
*M. episquamalis*^18^	*C. ramada*, *M. cephalus*	Scales	Off Japan, Egypt	8.6 ± 0.2 × 6.8 ± 0.1	4.4 × 2.2	–	Present	1.26
*M. exiguus*^15^	*C. ramada*, possibly also in *C. auratus*, *C. saliens*, *C. labrosus* and *M. cephalus*	Visceral peritoneum	France, Tunisia, Portugal	9.3 ± 0.6 × 8.2 ± 0.5	4.8 ± 0.2 × 2.8 ± 0.3	5	Absent	1.13
*M. hepatobiliaris*^10^	*C. ramada*	Liver and gall bladder	River Minho, Portugal	6.6 ± 0.3 × 5.20.3	3.0 ± 0.2 × 1.7 ± 0.2	4	Present	1.27
*M. labrosus*^10^	*C. labrosus*	Urinary bladder	River Minho, Portugal	10 ± 0.2 × 8.1 ± 0.3	4.5 ± 0.2 × 2.5 ± 0.2	5–7	Present	1.23
*M. mugauratus*^19^	*C. auratus*	Abdominal serosa	Black Sea off Ukraine	6.5 × 5.0	4.0 × 3.0	–	Absent	1.3
*M. mugchelo*^23^	*C. ramada*, *C. labrosus*	Gills or mesentery	Off Italy	6.06 ± 0.4 × 3.48 ± 0.9	2.19 ± 0.5 × 1.59 ± 0.3	5–6	Absent	1.74
*M. muscularis*^10^	*C. ramada*	Skeletal and heart muscle	River Minho, Portugal	9.1 ± 0.6 × 7.0 ± 0.6	4.3 ± 0.3 × 2.7 ± 0.2	5–6	Present	1.3
*M. parsi*^21^	*C. parsia*	Gills	India	9.1 × 8.1	4.4 × 2.8	5	Present	1.12
*M. parenzani*^16^	*C. labrosus*	Gills	Off Italy	5.4 × 5.4	~ 2	–	–	1.0
*M. parvus*^13^	*M. cephalus, C. auratus, C. saliens, P. haematocheila*	Gills, kidney, liver, mesentery, gall bladder, intestine, lower jaw	China, Ukraine, Black Sea, Indian Ocean	6.5–7.0 × 5.5–6.0	3.8–4.2 × 2.0	6–7	–	1.17
*M. peritonaeum*^10^	*C. labrosus*	Visceral peritoneum	River Minho, Portugal	8.1 ± 0.2 × 7.1 ± 0.2	3.8 ± 0.2 × 2.4 ± 0.2	4–5	Present	1.14
*M. pharyngobranchialis*^10^	*C. ramada*	Pharyngobranchial organ	River Minho, Portugal	9.3 ± 0.4 × 7.7 ± 0.4	4.7 ± 0.3 × 2.9 ± 0.2	6–7	Present	1.20
*M. ramadus*^10^	*C. ramada*	Gill lamellae	River Minho, Portugal	8.2 ± 0.5 × 7.9 ± 0.2	4.2 ± 0.2 × 3.0 ± 0.2	5–6	Absent	1.03
*M. renalis*^10^	*C. ramada*	Kidney	River Minho, Portugal	6.7 ± 0.2 × 5.8 ± 0.2	3.1 ± 0.2 × 1.9 ± 0.2	4	Present	1.15

The myxospores of *M. pupkoi* n. sp. are characterized by a sub-spherical to spherical shape in valvular view and an ellipsoidal shape in sutural view. Both polar capsules are equal in size and pyriform shaped, arranged side by side. The new species presents sutural folds all over the rim of the myxospore, and hence differs from *M. adeli*, *M. exiguus*, *M. mugauratus*, *M. mugchelo,* and *M. ramadus* spores, for which this feature is absent. The size of the novel myxospore is 5.84 ± 0.13 × 5.41 ± 0.09 µm and hence differs from *M. adiposus* (9.1 ± 0.3 × 9.0 ± 0.3 µm), *M. cerveirensis* (8.1 ± 0.2 × 6.8 ± 0.2 µm), *M. labrosus* (10.2 ± 0.2 × 8.1 ± 0.3 µm), *M. muscularis* (9.1 ± 0.6 × 7.0 ± 0.6 µm), *M. parsi* (9.1 × 8.1 µm), *M. peritonaeum* (8.1 ± 0.2 × 7.1 ± 0.2 µm), and *M. pharyngobranchialis* (9.3 ± 0.4 × 7.7 ± 0.4 µm), which have much larger spores. The new species is sub-spherical to spherical in shape, and hence differs from *M. adeli,* which is spindle shaped. The infection site of *M. pupkoi* n. sp. is the gill arch and hence it differs from *M. episquamalis,* which infects scales*.* It also differs from *M. cerveirensis*, *M. exiguus*, *M. hepatobiliaris*, and *M. parvus*, which infect various organs of the digestive track; and from *M. renalis*, which infects the kidney.

The new species is very similar in size to *M. parenzani,* which has been described to have round myxospores (~ 5.4 × 5.4 µm). However, it differs from the present species in having larger polar capsules. The polar capsules of *M. pupkoi* n. sp. are 2.4 ± 0.20 µm long and occupy about half of the myxospores. Conversely, the myxozpores of *M. parenzani* have been described to be ~ 2 µm long, to be positioned along the membrane (rather than side by side) and to only occupy the upper third of the spore. Although *M. pupkoi* n. sp. and *M. parenzani* infect the same host the morphological differences mentioned above justify the definition of a novel species.

### Phylogenetic analysis

**Figure 3:**
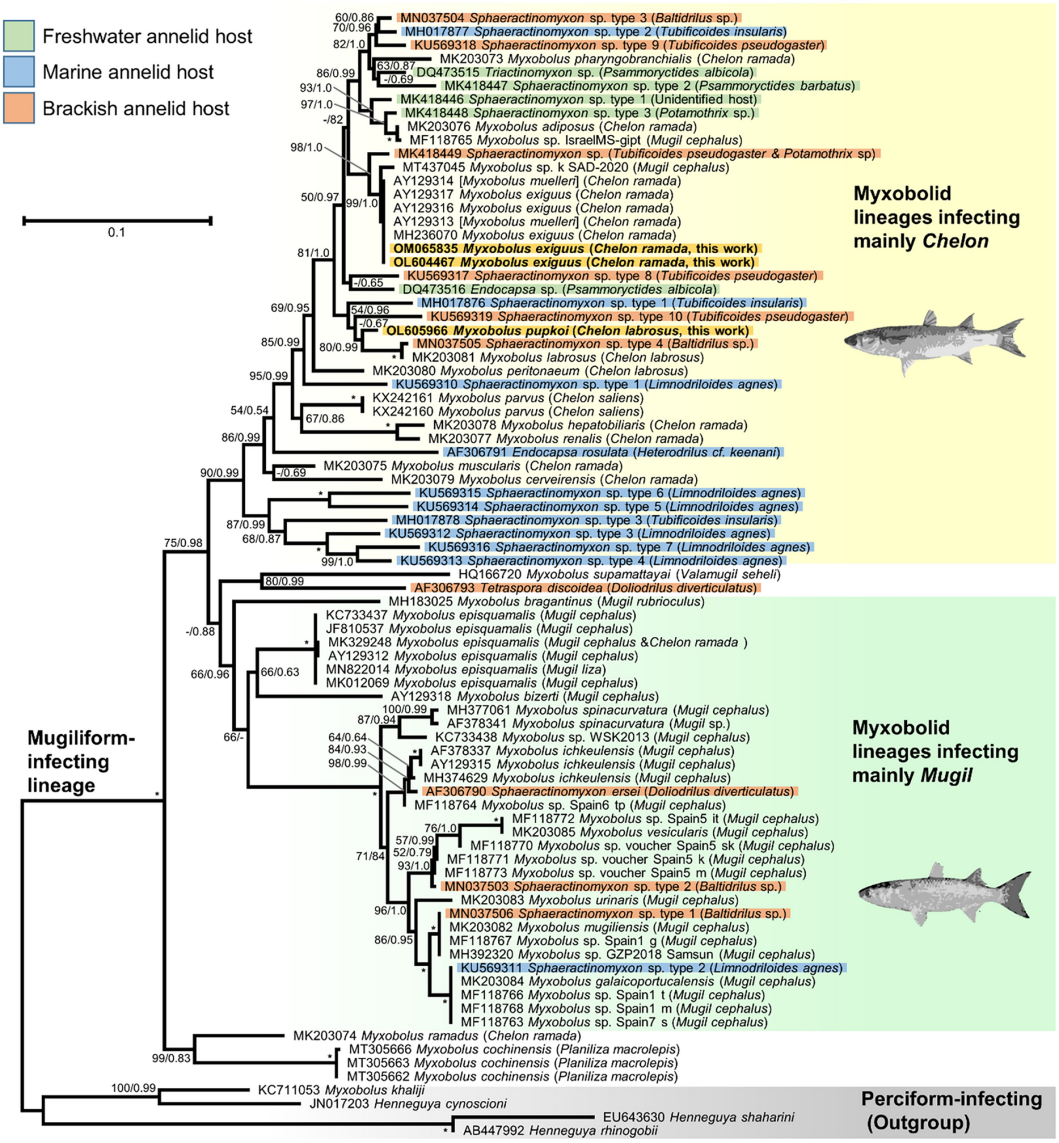
Phylogenetic relationships within the mugiliform-infecting lineage inferred from 18S rRNA sequences under the ML criterion (TVM + F + R3 model). The new sequences of *M. exiguus* (OL604467 and OM065835) and *M. pupkoi* (OL605966) are indicated in bold and with a yellow background. Branch supports (i.e. ML bootstrap percentages [BP] above 50/posterior probabilities [PP] above 0.5) are indicated near the corresponding nodes. Maximal support values (BP = 100/PP = 1.0) are indicated by an asterisk. A dash indicates BP < 50 or PP < 0.5. The sequences identified as *M. muelleri* in NCBI, but recognized to be *M. exiguus*^10,43^, are indicated within brackets.

The phylogenetic analyses included 79 sequences of the 18S rRNA gene from the mugiliform-infecting lineage of myxobolid, as well as four outgroup sequences. The trees reconstructed based on maximum likelihood and Bayesian criteria only differ in the position of a few low-supported branches (Fig. 3). These trees are in agreement with previous studies on myxozoans infecting mugilids^10,11,14^.

The phylogenetic tree divides the mugiliform-infecting lineage into two clades. The first harbors only *Myxobolus ramadus* and *Myxobolus cochinensis* (ML bootstrap support BP = 99; Bayesian posterior probability PP = 0.83)*.* The second, which includes most species (ML = 75; PP = 0.98), is composed of three subclades. One subclade includes myxobolid lineages, infecting mainly fish hosts from the genus *Chelon*, and is where *M. exiguus* and the newly described species *Myxobolus pupkoi* n. sp. branch. The second subclade includes myxobolid lineages infecting mainly fish hosts from the genus *Mugil.* Both subclades include myxozoans from annelid hosts, for which the fish host is unknown (Fig. 3). The third subclade includes *M. suppamattayai* and *Tetraspora discoidea* (ML = 80; PP = 0.99).

## Discussion

The present study describes *M. pupkoi* n. sp. and presents a novel tissue of infection, the gall bladder, for *M. exiguus*. It also reveals their presence in the Sea of Galilee. Morphologic and morphometric differences were observed between the myxospores of *M. exiguus* found in the visceral peritoneum and in the gall bladder, and their taxonomic identity could only be confirmed based on the 18S rRNA analysis. These data illustrate, once more, the importance of 18S rRNA sequences to complement the morphological identification of myxozoan species^9,10,19^, especially in the case of mugilids, which are infected with closely related myxobolid parasites^10^.

Tissue specificity is a characteristic of many histozoic myxozoans and an important taxonomical character^24–27^. Unfortunately, because of the small number of infected fish observed, the infected tissues were used for molecular identifications rather than histological preparation. Future work should determine, which tissue of the gill arch is the site of *M. pupkoi* infection (e.g., blood vessels, cartilaginous matrix or the connective tissue), following Molnár et al.^24,27^. *Myxobolus exiguus* is a histozoic parasite described from the visceral peritoneum^14^. Its presence in the gall bladder bile, which is a coelozoic localization, is thus surprising. However, it is possible that the plasmodia found in the bile originated from the gall bladder wall or the hepatic bile ducts and thus is of a histozoic origin^25,28^.

Both *M. exiguus* and *M. pupkoi* n. sp. belong to a well-defined clade of mugilid infecting myxozoans^14^. Members of this clade have also been found in marine, brackish and freshwater annelids present in estuaries^1,10,29^. Since mugilids are not native species from the Sea of Galilee and Jordan Valley system, it is most likely that these myxozoans were introduced to the Sea of Galilee with their mugilid hosts during stocking^5,30^, being the infection originated in the Mediterranean or the coastal plain estuaries of Israel from where the fingerlings originated before their introduction to the lake.

The fact that mugilid fingerlings are hosting numerous myxozoan parasites, sometimes even within a single individual, has been noted by Sharon et al.^11^. It was further suggested that the introduction of wild caught mugilid to new growing area may lead to the spread of their myxozoan parasites^11^. We here show that this prediction likely reflects the course of events that occurred in Israel where the transfer of infected fingerlings from the coastal environment led the presence of alien parasites in the Sea of Galilee.

Myxozoans need two hosts to complete their life cycle, it may thus seem that myxozoans are not a serious threat to the Sea of Galilee since the annelid hosts were not transferred with the fish hosts. However, the establishment of a transmission cycle in that environment using local annelids species cannot be ruled out.

The annelid fauna of the Sea of Galilee is poorly known. Previous studies have indicated the presence of *Psammoryctides albicola* as well as members of the genus *Potamothrix* (previously identified as *Euilyodrilus*)^30^. These freshwater annelids are known to harbor myxozoans closely related to the ones described in this work^10^ (see Fig. 3). This suggests that the repeated introduction of infected mugillid fingerlings to the Sea of Galilee could have led to the establishment of myxozoan parasites to this new environment. While we did not find myxozoan parasites on the *M. cephalus* specimen studied during this work, their past introductions from wild-caught fingerlings pose the same threats than *C. ramada*^2–5^. Indeed, *M. cephalus* fingerlings from the north of Israel were found to be infected by another myxobolid species (sequence MF118765, Fig. 3)^11^, which has been considered to be *M. adiposus*^10^*.* This observation suggests that more than two myxobolid species may have been introduced to the Sea of Galilee with their mugilid hosts. Since 2018, only farm raised fingerlings of *M. cephalus* have been introduced to the Sea of Galilee. These fingerlings are expected to be parasite-free. If the introduced myxozoans cannot reproduce due to the lack of annelid hosts, we expect to stop observing myxobolid infected mugilids in the future. We thus recommend to closely monitor the presence of these parasites in the next 5 years to determine if these myxozoan parasites disappear.

## Methods

### Parasite material and morphological identification

*Mugil cephalus* specimens (n = 10) with a length of 18–20 cm, and *Chelon ramada* specimens (n = 23) with a length of 15–20 cm freshly collected from the Sea of Galilee, were obtained from a local fisherman and immediately transported to the lab. The fish sampling was performed under the authorization of the Fisheries and Aquaculture Department of the Israeli Ministry of Agriculture and Rural Development (authorization provided on 11.11.2020). The fish specimens were carefully examined externally for the presence of plasmodia followed by a dissection of each fish sample. Gills, scales, brain, kidney, visceral peritoneum, and other body parts were removed and examined under a stereo-microscope. When plasmodia were identified, fresh myxospores were photographed under a compound microscope with a DS-Ri2 Nikon photographic unit. The myxospores were stained with freshly prepared Ziehl–Neelsen and Giemsa solutions^31^. Spore measurements were done using a calibrated ocular micrometer.

### DNA isolation and amplification

The samples for molecular work were fixed in absolute alcohol and stored at −20 °C. The extraction of genomic DNA was performed using the Qiagen DNeasy Blood and Tissue Kit according to the manufacturer’s instructions. PCR amplification of the 18S rRNA was performed using both universal and myxosporean specific primers^32–34^ (Table 3). The 25 µL of PCR mix consisted of 1 μL DNA template (9.9 ng/μL of *M. exiguus* and 34.7 ng/μL of *M. pupkoi*), 2.5 μL of 10X Taq buffer (Takara Bio Inc., Japan), 0.2 μL of Ex Taq polymerase (5 units/μL; Takara Bio Inc., Japan), 2 μL of 20 µM dNTP mix (Takara Bio Inc., Japan), 2.5 μL of 5 pmol/μL of each primer (Merck, Germany), 0.2 μL of DMSO (100%, Sigma Aldrich, US), 5 μL of 5 M Betaine (Bio-Lab Ltd., Jerusalem), and 9.1 μL of molecular grade water. Amplification was performed using the following protocol: 1—initial denaturation at 94 °C for 5 min; 2—35 cycles of denaturation at 94 °C for 45 s, annealing of primers at 58 °C for 45 s, and extension at 72 °C for 2 min; 3—a final extension at 72 °C for 10 min. The obtained PCR products were purified using the ExoSAP method^35^.

**Table 3:** PCR primers used for the amplification and sequencing of the 18S rRNA gene.

Name	Sequence (5′–3′)	Usage	Source
ER1B1	ACCTGGTTGATCCTGCCAG	PCR sequencing	^44^
ER1B10	CTTCCGCAGGTTCACCTACGG	PCR sequencing	^44^
Myxgen4F	GTGCCTTGAATAAATCARAG	Sequencing	^33^
Myxgen4R	CTYTGATTTATTCAAGGCAC	Sequencing	^33^
Myx4F	GTTCGTGGAGTGATCTGTCAG	Sequencing	^32^
Myx4R	CTGACAGATCACTCCACGAAC	Sequencing	^34^
ACT1F	TGGCAGCGAGAGGTGAAATT	Sequencing	^34^
ACT1R	AATTTCACCTCTCGCTGCCA	Sequencing	^34^

Sequencing was performed using the external primers ER1B1 and ER1B10 together with six additional primers (Table 3) at the DNA Sequencing Unit at Tel-Aviv University on an ABI 3500xl Genetic Analyzer (Applied Biosystems™). The obtained sequences were visualized, assembled and edited using Geneious 11.1.5.

### Phylogenetic reconstructions

To reconstruct the phylogenetic relationships of the Israeli species, we first blasted the obtained 18S rRNA sequences, against the NCBI nr databased (https://blast.ncbi.nlm.nih.gov/) on 05.09.2021. This allowed us to determine that both sequences belong to a distinct lineage of myxobolid infecting mugilids in the Histozoic III lineage sensus^14,36^. We downloaded all sequence hits from this clade that were longer than 800 bp. The sequences of *Henneguya shaharini* (EU643630), *H. rhinogobii* (AB447992), *H. cynoscioni* (JN017203), and *Myxobolus khaliji* (KC711053) were used as outgroups.

Because the start and end of sequences can include sequencing errors, we excluded the first and last 10 base pairs of all sequences, except for sequence MH183025 *Myxobolus bragantinus* for which we removed the first 20 bp based on a manual examination. The webserver Guidance2^37^ (last accessed 15.12.2021) was used to align the edited sequences and to remove ambiguously aligned positions. Specifically, the sequences were aligned with the MAFFT algorithm under the options “Max-Iterate: 1000” and “Pairwise Alignment Method: localpair”. Positions with a score below 0.93 were removed as well as positions with more than 50% of missing data. Finally, we also removed positions corresponding to the annealing regions of the primers 18Se (5′-CTGGTTGATCCTGCCAGT-3′)^38^ and 18Sr (5′-CTACGGAAACCTTGTTACG-3′)^39^, the primers used to amplify most sequences in our sequence alignment^10,14,40^. The final dataset included 83 taxa and 1781 positions (Supplementary Files 1 and 2).

Phylogenetic relationships were reconstructed both under the maximum likelihood and the Bayesian criteria. Maximum likelihood analyses were performed with the program IQ-TREE version 1.6.12^41^. The analyses were run with the options –m MFP –b 1000 (i.e., ModelFinder + tree reconstruction + 1000 non-parametric bootstrap replicates). The model selected based on the BIC criterion was the TVM + F + R3 model. Bayesian reconstructions were performed with PhyloBayes MPI version 1.8^42,45^ under the CAT + GTR + GAMMA 4 model. Four chains ran for 55,000 points. The first 5000 trees from each chain were discarded as burn-in, and the consensus was built from trees sampled every 10 points (i.e., 5000 trees per chain). At the end of the run, the bpcomp maxdiff value was 0.051, indicating a proper convergence. We also verified that, for all parameters, the tracecomp eff size values were above 5000 and the rel_diff below 0.05, as recommended by the PhyloBayes manual.

### ZooBank

This published work and the Nomenclatural Acts for Myxobolus pupkoi n. sp., have been registered in ZooBank. The ZooBank Life Science Identifier (LSID) for this publication is: http://zoobank.org/urn:lsid:zoobank.org:act:ED379E2F-4522-4478-B13B-D444E1F6E66D.

## Supplementary Information

Supplementary Information 1.

Supplementary Information 2.

## Data Availability

All the uncropped images of *M. exiguus* and *M. pupkoi* n. sp. are available in the Figshare repository (10.6084/m9.figshare.17705105). Novel sequences obtained in this study were deposited to GenBank under accession numbers OM065835 and OL604467 for *M. exiguus* and OL605966 for *M. pupkoi*.
