# Author Correction: Myxozoan infection in thinlip mullet *Chelon ramada *(Mugiliformes: Mugilidae) in the Sea of Galilee

**DOI:** 10.1038/s41598-023-31318-z

**Published:** 2023-03-20

**Authors:** Aditya Gupta, Michal Haddas-Sasson, Kfir Gayer, Dorothée Huchon

**Affiliations:** 1 School of Zoology, George S. Wise Faculty of Life Sciences, Tel Aviv University, 6997801 Tel-Aviv, Israel; 2 Steinhardt Natural History Museum, Tel Aviv University, 6997801 Tel-Aviv, Israel

Correction to: *Scientific Reports* 10.1038/s41598-022-13215-z, published online 16 June 2022

The original version of this Article contained errors.

Illumina sequencing of the DNA extract of *Myxobolus pupkoi* led us to discover that the host of this species is not *Chelon ramada,* but rather *C. labrosus*. The morphological identification error stemmed from the fact that *C. labrosus* is extremely rare in the Sea of Galilee, and thus its identification was overlooked. We only sequenced the COI gene of a single fish specimen among the 23 fish sampled to confirm the morphological identification. Unlike *C. ramada*, *C. labrosus* was never voluntarily introduced to the Sea of Galilee, but rather was a hitchhiker introduced together with *C. ramada*^1^. In absence of reproduction in the lake, its population size is very small^1^.

We assembled the complete mitochondrial genome of *C. labrosus* from the Illumina data obtained from the sequencing of the *M. pupkoi* sample (deposited under accession OX417109). We also confirmed using PCR amplifications that the fish host of *M. exiguus* studied in our work is *C. ramada* (deposited under accessions OX417110-1).

This misidentification that we here correct does not affect the main conclusions of our manuscript. Like *C. ramada, C. labrosus* is an alien species and its infection took place in the Mediterranean Sea, where the fingerlings were caught. Additionally, the differences in spore morphology between *M. pupkoi* and other *Myxobolus* parasites of the genus *Chelon* warrant the description of a new species. The authors apologize for the misidentification and any confusion caused.

Corresponding text modifications are indicated below.

In the Abstract,

“These catadromous species do not reproduce in the lake, consequently, fingerlings have been introduced every year since 1958. Following a survey of myxozoan infections in the Sea of Galilee, we described *Myxobolus pupkoi* n. sp. infecting the gill arches, and reported *Myxobolus exiguus* from visceral peritoneum and gall bladder of *C. ramada*. The prevalence of infection of both *Myxobolus pupkoi* n. sp. and *M. exiguus* were 11.5% (2/23). Our study indicates that the parasites infecting *C. ramada* belong to a lineage of myxozoans infecting mugilids”

should read:

“These catadromous species do not reproduce in the lake, consequently, fingerlings have been introduced every year since 1958. Few additional mugilid species have been introduced unintentionally together with these two species, including *C. labrosus*. Following a survey of myxozoan infections in the Sea of Galilee, we described *Myxobolus pupkoi* n. sp. infecting the gill arches of *C. labrosus*, and reported *Myxobolus exiguus* from visceral peritoneum and gall bladder of *C. ramada*. Our study indicates that the parasites infecting *C. ramada* and *C. labrosus* belong to a lineage of myxozoans infecting mugilids.”

In the Results section, under the subheading ‘*Myxobolus exiguus* Thélohan, 1895 from the Sea of Galilee’,

“Prevalence of infection: 11.5% (02/23)”

should read:

Because of fish misidentification the prevalence of infection cannot be computed.

In the Results section, under subheading ‘*Myxobolus pupkoi* n. sp.’,

“Type host: *Chelon ramada* (Risso, 1827), vern. thinlip mullet, Family: Mugilidae.”

should read:

“Type host: *Chelon labrosus* (Risso, 1827), vern. thicklip grey mullet, Family: Mugilidae.”

“Prevalence of infection: 11.5% (02/23).”

should read:

Because of fish misidentification the prevalence of infection cannot be computed.

“*M. pupkoi* n. sp. and *M. parenzani* infect different hosts: *C. ramada* and *C. labrosus*, respectively.”

should read:

“Although *M. pupkoi* n. sp. and *M. parenzani* infect the same host the morphological differences mentioned above justify the definition of a novel species.”

Additionally, in Figure 2 legend,

“*Myxobolus pupkoi* n. sp. parasite of the gill arch of *Chelon ramada*.”

should read:

“*Myxobolus pupkoi* n. sp. parasite of the gill arch of *Chelon labrosus*.”

Finally, the original Figure 3 and Table 2 showing the incorrect host name appear below as Figure 3 and Table 2, respectively.

**Figure 3:**
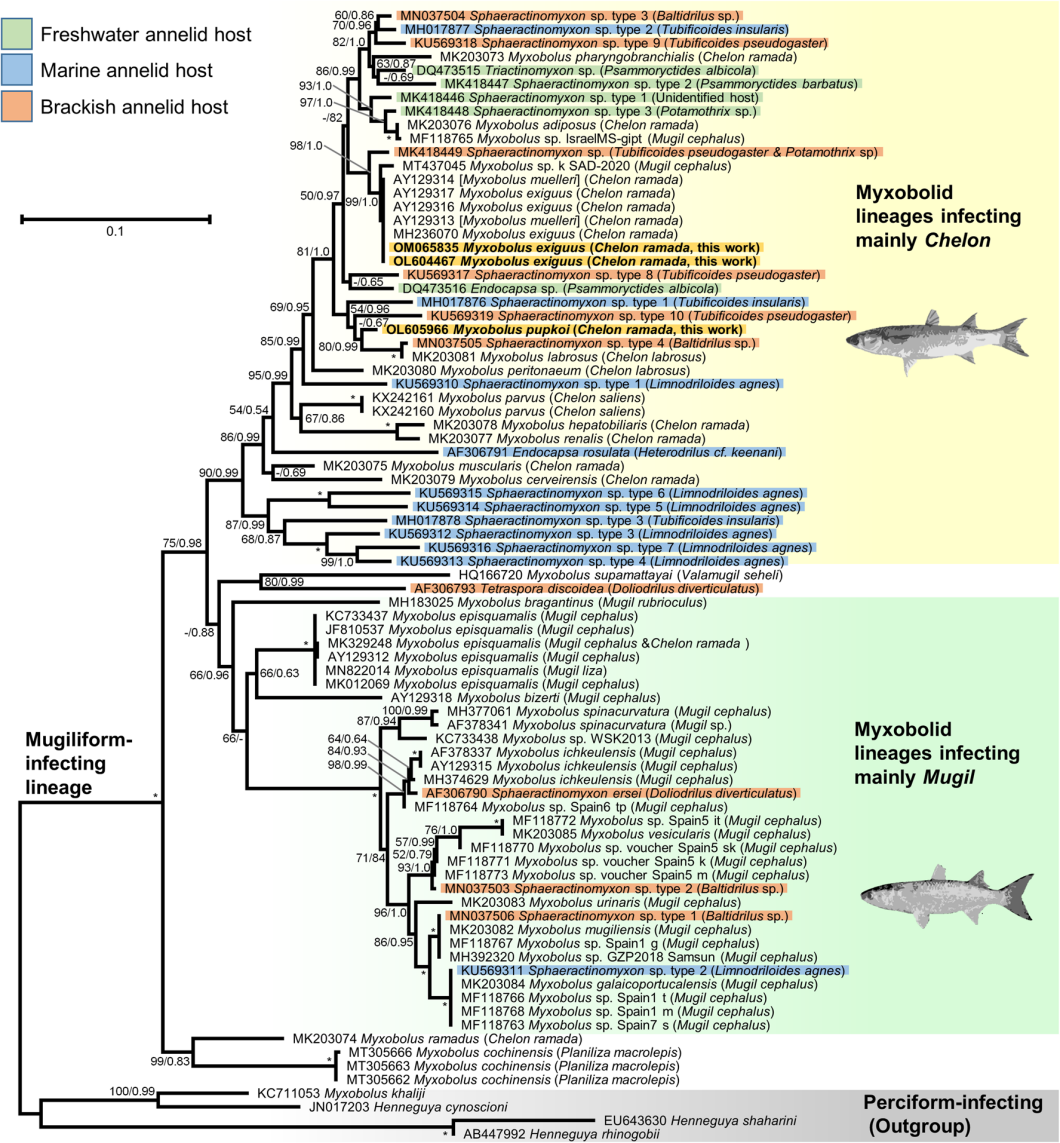
Phylogenetic relationships within the mugiliform-infecting lineage inferred from 18S rRNA sequences under the ML criterion (TVM + F + R3 model). The new sequences of *M. exiguus* (OL604467 and OM065835) and *M. pupkoi* (OL605966) are indicated in bold and with a yellow background. Branch supports (i.e. ML bootstrap percentages [BP] above 50/posterior probabilities [PP] above 0.5) are indicated near the corresponding nodes. Maximal support values (BP = 100/PP = 1.0) are indicated by an asterisk. A dash indicates BP < 50 or PP < 0.5. The sequences identified as *M. muelleri* in NCBI, but recognized to be *M. exiguus*^10,43^, are indicated within brackets.

**Table 2:** Comparative description of *Myxobolus pupkoi* n. sp. with myxobolid species infecting *Chelon ramada* (measurements in micrometer).

Species	Host	Site of infection	Locality	Myxospores	Polar capsule	No. of coils	Parietal folds	LS/WS ratio
*Myxobolus pupkoi* n. sp. (present study)	*Chelon ramada*	Gill arch	Sea of Galilee, Israel	5.84 ± 0.13 × 5.41 ± 0.09	2.44 ± 0.20 × 1.34 ± 0.19	4–5	Present	1.07
*M. exiguus* (present study)	*C. ramada*	Gall bladder	Sea of Galilee, Israel	7.80 ± 0.35 × 6.60 ± 0.24	3.79 ± 0.13 × 2.54 ± 0.07	3–4	Absent	1.18
*M. exiguus* (present study)	*C. ramada*	Visceral peritoneum	Sea of Galilee, Israel	6.63 ± 0.25 × 6.01 ± 0.30	3.41 ± 0.19 × 2.16 ± 0.10	3–4	Absent	1.10
*M. adeli*^8^	*C. auratus*	Digestive tract, swim bladder, gills, muscle	Mediterranean Sea off Spain, Azov and Black Sea	6.2 ± 0.3 × 7.2 ± 0.3	3.1 ± 0.3 × 1.8 ± 0.2	4	Absent	0.86
*M. adiposus*^10^	*C. ramada*	Adipose tissue	River Minho, Portugal	9.1 ± 0.3 × 9.0 ± 0.3	4.6 ± 0.3 × 3.0 ± 0.3	6–7	Present	1.01
*M. cerveirensis*^10^	*C. ramada*	Intestine	River Minho, Portugal	8.1 ± 0.2 × 6.8 ± 0.2	4.2 ± 0.2 × 2.8 ± 0.2	4–5	Present	1.19
*M. episquamalis*^18^	*C. ramada*, *M. cephalus*	Scales	Off Japan, Egypt	8.6 ± 0.2 × 6.8 ± 0.1	4.4 × 2.2	–	Present	1.26
*M. exiguus*^15^	*C. ramada*, possibly also in *C. auratus*, *C. saliens*, *C. labrosus* and *M. cephalus*	Visceral peritoneum	France, Tunisia, Portugal	9.3 ± 0.6 × 8.2 ± 0.5	4.8 ± 0.2 × 2.8 ± 0.3	5	Absent	1.13
*M. hepatobiliaris*^10^	*C. ramada*	Liver and gall bladder	River Minho, Portugal	6.6 ± 0.3 × 5.20.3	3.0 ± 0.2 × 1.7 ± 0.2	4	Present	1.27
*M. labrosus*^10^	*C. labrosus*	Urinary bladder	River Minho, Portugal	10 ± 0.2 × 8.1 ± 0.3	4.5 ± 0.2 × 2.5 ± 0.2	5–7	Present	1.23
*M. mugauratus*^19^	*C. auratus*	Abdominal serosa	Black Sea off Ukraine	6.5 × 5.0	4.0 × 3.0	–	Absent	1.3
*M. mugchelo*^23^	*C. ramada*, *C. labrosus*	Gills or mesentery	Off Italy	6.06 ± 0.4 × 3.48 ± 0.9	2.19 ± 0.5 × 1.59 ± 0.3	5–6	Absent	1.74
*M. muscularis*^10^	*C. ramada*	Skeletal and heart muscle	River Minho, Portugal	9.1 ± 0.6 × 7.0 ± 0.6	4.3 ± 0.3 × 2.7 ± 0.2	5–6	Present	1.3
*M. parsi*^21^	*C. parsia*	Gills	India	9.1 × 8.1	4.4 × 2.8	5	Present	1.12
*M. parenzani*^16^	*C. labrosus*	Gills	Off Italy	5.4 × 5.4	~ 2	–	–	1.0
*M. parvus*^13^	*M. cephalus, C. auratus, C. saliens, P. haematocheila*	Gills, kidney, liver, mesentery, gall bladder, intestine, lower jaw	China, Ukraine, Black Sea, Indian Ocean	6.5–7.0 × 5.5–6.0	3.8–4.2 × 2.0	6–7	–	1.17
*M. peritonaeum*^10^	*C. labrosus*	Visceral peritoneum	River Minho, Portugal	8.1 ± 0.2 × 7.1 ± 0.2	3.8 ± 0.2 × 2.4 ± 0.2	4–5	Present	1.14
*M. pharyngobranchialis*^10^	*C. ramada*	Pharyngobranchial organ	River Minho, Portugal	9.3 ± 0.4 × 7.7 ± 0.4	4.7 ± 0.3 × 2.9 ± 0.2	6–7	Present	1.20
*M. ramadus*^10^	*C. ramada*	Gill lamellae	River Minho, Portugal	8.2 ± 0.5 × 7.9 ± 0.2	4.2 ± 0.2 × 3.0 ± 0.2	5–6	Absent	1.03
*M. renalis*^10^	*C. ramada*	Kidney	River Minho, Portugal	6.7 ± 0.2 × 5.8 ± 0.2	3.1 ± 0.2 × 1.9 ± 0.2	4	Present	1.15

The original Article has been corrected.
