# Correction: Myxozoan infection in thinlip mullet *Chelon ramada* (Mugiliformes: Mugilidae) in the Sea of Galilee

**DOI:** 10.1038/s41598-025-22417-0

**Published:** 2025-10-13

**Authors:** Aditya Gupta, Michal Haddas-Sasson, Kfir Gayer, Dorothée Huchon

**Affiliations:** 1 School of Zoology, George S. Wise Faculty of Life Sciences, Tel Aviv University, 6997801 Tel-Aviv, Israel; 2 Steinhardt Natural History Museum, Tel Aviv University, 6997801 Tel-Aviv, Israel

Correction to: *Scientific Reports* 10.1038/s41598-022-13215-z, published online 16 June 2022

The original version of this Article did not include evidence of ZooBank registration details, as required by Article 8.5.3 of the International Code of Zoological Nomenclature (ICZN)^1^.

This published work and the Nomenclatural Acts for *Myxobolus pupkoi* n. sp., have been registered in ZooBank. The ZooBank Life Science Identifier (LSID) for this publication is: http://zoobank.org/urn:lsid:zoobank.org:act:ED379E2F-4522-4478-B13B-D444E1F6E66D

The original Article has been corrected.
